# Targeting screening and treatment for latent tuberculosis infection towards asylum seekers from high-incidence countries – a model-based cost-effectiveness analysis

**DOI:** 10.1186/s12889-021-12142-4

**Published:** 2021-11-26

**Authors:** Florian M. Marx, Barbara Hauer, Nicolas A. Menzies, Walter Haas, Nita Perumal

**Affiliations:** 1 Department for Infectious Disease Epidemiology, Respiratory Infections Unit, Robert Koch Institute, Berlin, Germany; 2 Department of Paediatrics and Child Health, Desmond Tutu TB Centre, Faculty of Medicine and Health Sciences, Stellenbosch University, Cape Town, South Africa; 3 DSI-NRF South African Centre of Excellence in Epidemiological Modelling and Analysis (SACEMA), Stellenbosch University, Stellenbosch, South Africa; 4 Department of Global Health and Population, Harvard T.H. Chan School of Public Health, Boston, USA; 5 Immunization Unit, Department for Infectious Disease Epidemiology, Robert Koch Institute, Berlin, Germany

## Abstract

**Background:**

Enhancing tuberculosis (TB) prevention and care in a post-COVID-19-pandemic phase will be essential to ensure progress towards global TB elimination. In low-burden countries, asylum seekers constitute an important high-risk group. TB frequently arises post-immigration due to the reactivation of latent TB infection (LTBI). Upon-entry screening for LTBI and TB preventive treatment (TPT) are considered worthwhile if targeted to asylum seekers from high-incidence countries who usually present with higher rates of LTBI. However, there is insufficient knowledge about optimal incidence thresholds above which introduction could be cost-effective. We aimed to estimate, among asylum seekers in Germany, the health impact and costs of upon-entry LTBI screening/TPT introduced at different thresholds of country-of-origin TB incidence.

**Methods:**

We sampled hypothetical cohorts of 30–45 thousand asylum seekers aged 15 to 34 years expected to arrive in Germany in 2022 from cohorts of first-time applicants observed in 2017–2019. We modelled LTBI prevalence as a function of country-of-origin TB incidence fitted to data from observational studies. We then used a probabilistic decision-analytic model to estimate health-system costs and quality-adjusted life years (QALYs) under interferon gamma release assay (IGRA)-based screening for LTBI and rifampicin-based TPT (daily, 4 months). Incremental cost-effectiveness ratios (ICERs) were calculated for scenarios of introducing LTBI screening/TPT at different incidence thresholds.

**Results:**

We estimated that among 15- to 34-year-old asylum seekers arriving in Germany in 2022, 17.5% (95% uncertainty interval: 14.2–21.6%) will be latently infected. Introducing LTBI screening/TPT above 250 per 100,000 country-of-origin TB incidence would gain 7.3 (2.7–14.8) QALYs at a cost of €51,000 (€18,000–€114,100) per QALY. Lowering the threshold to ≥200 would cost an incremental €53,300 (€19,100–€122,500) per additional QALY gained relative to the ≥250 threshold scenario; ICERs for the ≥150 and ≥ 100 thresholds were €55,900 (€20,200–€128,200) and €62,000 (€23,200–€142,000), respectively, using the next higher threshold as a reference, and considerably higher at thresholds below 100.

**Conclusions:**

LTBI screening and TPT among 15- to 34-year-old asylum seekers arriving in Germany could produce health benefits at reasonable additional cost (with respect to international benchmarks) if introduced at incidence thresholds ≥100. Empirical trials are needed to investigate the feasibility and effectiveness of this approach.

## Background

Progress towards the global targets for eliminating tuberculosis (TB), an infectious disease that claimed an estimated 1.4 million lives in 2019, is seriously undermined by the concurrent COVID-19 pandemic and associated measures for its containment. Increases in the TB burden due to lockdowns and disruptions of healthcare service delivery have been predicted [1, 2]. Enhanced efforts to prevent, detect and treat TB will therefore be necessary to protect those affected by the disease and ensure progress towards the global targets for TB elimination [2].

In countries with a low TB burden, immigrants including asylum seekers carry a disproportionate burden of TB and therefore represent a key target group for prevention and care strategies [3]. Guidelines and practice of focused TB interventions among immigrants vary widely among countries [4, 5]. Traditionally, most countries rely on screening for active TB upon entry, most commonly by means of chest radiography (CXR) examination, alone or with clinical evaluation [4]. The purpose is to identify immigrants with active TB promptly, in order to initiate treatment and prevent onward transmission. Major concerns, however, include high costs of untargeted screening, the limited accuracy of available tests, and the relatively low screening yield especially among immigrants from countries with low TB incidence [6, 7].

A considerable burden of incident TB among immigrants arises in the years following immigration [8–11], likely due to the reactivation of latent TB infection (LTBI) [11], possibly exceeding the burden of active TB detectable upon entry [9]. Some countries therefore focus primarily on screening for *Mycobacterium tuberculosis* (*M. tb*) infection [5, 12]. The advantage of this approach is that individuals who test positive for infection can be evaluated and treated for either LTBI or TB disease. Studies suggest that screening for *M. tb* infection among immigrants and providing TB preventive treatment (TPT) to those with LTBI may be cost-effective for TB control depending on local contexts [13–15]. Children, adolescents and young adults should be prioritized for LTBI screening because in older adults the risks imposed by TPT drug-adverse reactions may outweigh its benefits [14].

Targeting LTBI screening and TPT towards individuals arriving from countries with higher TB incidence has been recommended as these are expected to have a higher prevalence of LTBI [15, 16]. However, there is currently insufficient knowledge about optimal thresholds of country-of-origin TB incidence that should be considered as ‘high incidence’, i.e. above which introducing LTBI screening and TPT could produce significant health benefits at reasonable costs.

Here, we present a model-based cost-effectiveness analysis of upon-entry screening among asylum seekers arriving in Germany, the country with the highest number of asylum seekers in the European Union (EU) in recent years [17]. Rates of incident TB in the years after immigration appear to be high [9, 18], echoing findings from studies elsewhere in Europe [8, 10, 11], and highlighting the need to explore preventive strategies among asylum seekers [12, 19].

We aimed to estimate the health impact and costs of LTBI screening and TPT among asylum seekers of adolescent and young adult age (15 to 34 years) if implemented alongside the current mandatory CXR-based screening policy. A targeted approach was assumed, under which asylum seekers would be eligible for LTBI screening and TPT if they originated from a country with a high TB incidence. We aimed to explore the cost-effectiveness at different thresholds at which countries of origin would be considered ‘high incidence’ and asylum seekers would thus be eligible for LTBI screening and TPT.

## Methods

### Study setting

Germany is the EU country with the largest population (83.2 million in 2020). In 2015/2016, approximately 1.16 million first-time asylum applicants were registered, accounting for 47% of applications in the European Union [17]. This number declined to 0.50 million in the years 2017–2019. More than 80% of asylum seekers are children, adolescents and young adults aged < 35 years [17]. Asylum seekers arriving in Germany routinely undergo mandatory screening for active TB in accordance with the German Protection Against Infection Act (*IfSG*), regardless of country of origin, before being admitted to common housing/reception centers. Children, young adolescents aged < 15 years and pregnant women are primarily evaluated for *M. tb* infection either via tuberculin skin test (TST) or interferon gamma release assay (IGRA), whereas asylum seekers aged 15 years and older (who are not pregnant) are screened for TB disease via clinical and CXR examination. Evaluation for *M. tb* infection and TPT are recommended for the management of individuals exposed to infectious TB [20]. LTBI screening and TPT are currently not routinely offered to asylum seekers.

### Study design

**Fig. 1:**
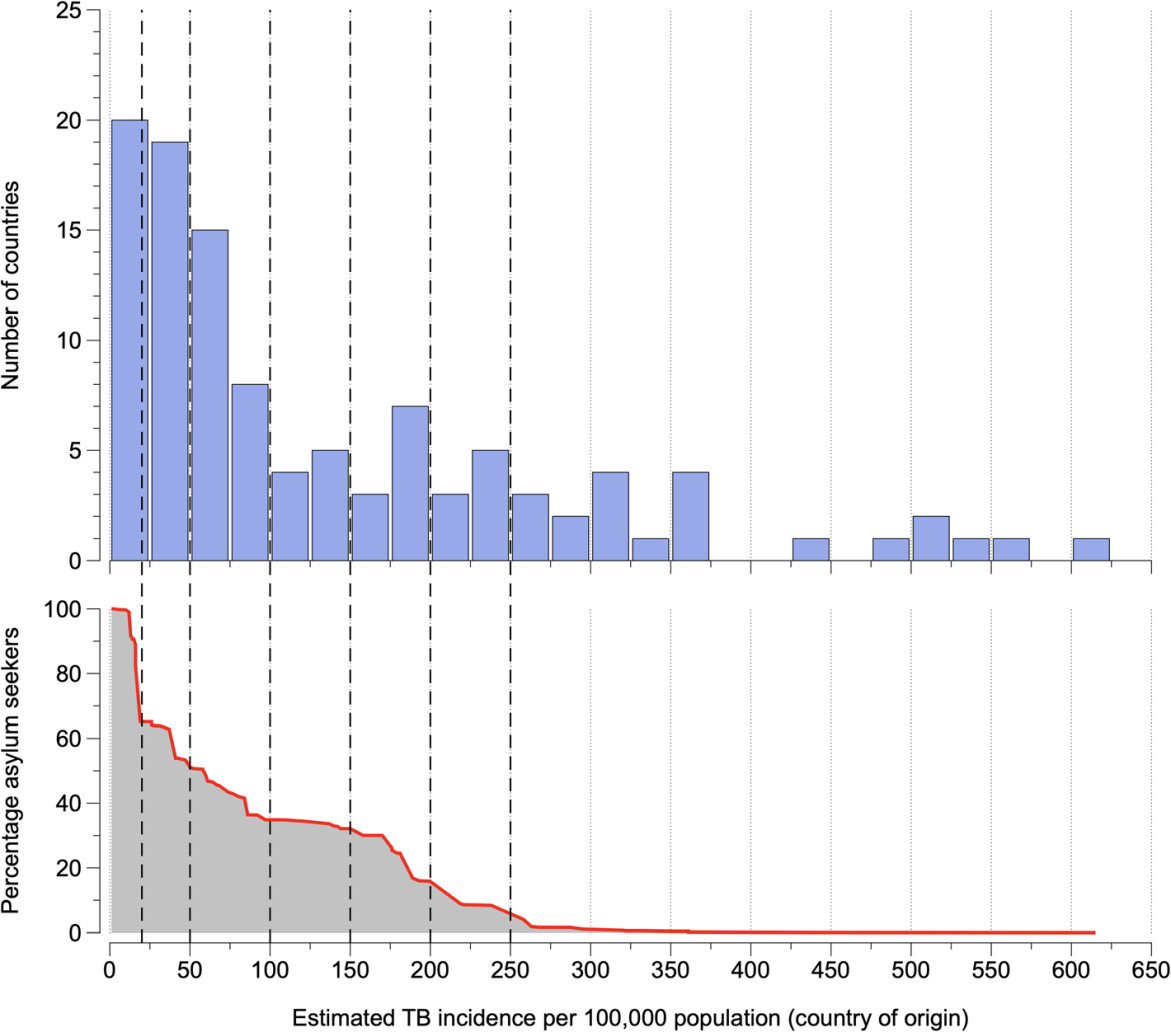
Cohort of asylum seekers in Germany 2017-2019. Blue bars show asylum seekers’ countries of origin by estimated TB incidence; dashed vertical lines show the country-of-origin TB incidence thresholds investigated in this study above which asylum seekers would be eligible for latent TB infection (LTBI) screening; At a TB incidence threshold X, asylum seekers are eligible for screening and treatment of LTBI if their country of origin’s estimate of TB incidence exceeds the threshold X TB cases per 100,000 population. The red line shows cumulative percentages of asylum seekers who would be eligible for LTBI screening at a given incidence threshold.

We developed a probabilistic decision-analytic model using R statistical software (version 4.0.3). The model draws multiple random samples from the population of 15- to 34-year-old asylum applicants from 110 countries of origin who were registered in Germany between 2017 and 2019 [17] (Fig. 1), with sampling probability proportional to the number registered per country of origin. The model then estimates the health impact and costs of LTBI screening and TPT.

We assumed that IGRA-based screening for *M. tb* infection would be introduced among 15- to 34-year-old asylum seekers in addition to clinical and CXR examinations for TB disease that are currently mandatory for all asylum seekers aged 15 years and above. We investigated different scenarios under which IGRA-based screening would be restricted to asylum seekers arriving from countries of origin above a specific TB incidence threshold, based on 2019 World Health Organization (WHO) incidence estimates [21]. Following visual exploration of the distribution of asylum seekers by country-of-origin TB incidence, we considered the following eligibility thresholds for country-of-origin estimated TB incidence, 0 (no threshold), 20, 50, 100, 150, 200, and 250 cases per 100,000 population.

Individuals who screen positive for *M. tb* infection (IGRA-positive), and in whom no diagnosis of active TB is established, would be considered latently infected and offered TPT. We assumed that TPT would consist of a short-course regimen of daily rifampicin (600 mg) for four months. The rationale for considering this regimen is that it is recommended and available for TPT in Germany [20] and by the WHO [22]. It is shorter and safer than isoniazid monotherapy (9 months), prompting hopes for higher initiation and completion rates. Key assumptions for the study are shown in the Box.

**(Box)** Main assumptions for the study.

**Table:** 

(1)	In 2022, between 30 and 45 thousand asylum seekers aged 15 to 34 years will arrive in Germany, a range extrapolated from recent trends prior to the COVID-19 pandemic [17]. Their countries of origin will be representative of those among asylum seekers of the same age who arrived during the years 2017 to 2019.
(2)	The prevalence of LTBI is calculated as a log-transformed linear function of TB incidence estimated for asylum seekers’ countries of origin, based on estimates (by country of origin) obtained from studies in Europe [23–26].
(3)	Among asylum seekers with LTBI, the development of active TB post entry is due to the reactivation of infection acquired prior to arrival in Germany. Between 2.5 and 8.0% of asylum seekers with untreated LTBI will experience reactivation within 20 years post-immigration [11, 27, 28].
(4)	Asylum seekers who are eligible (based on their age and country of origin) will receive interferon gamma release assay (IGRA)-based screening for LTBI. Those who screen positive, and in whom active TB disease was excluded through clinical and chest radiography-based examination, will be offered short-course TB preventive treatment (TPT) with 600 mg rifampicin daily for 4 months.
(5)	Of IGRA-positive individuals, 60–80% will initiate LTBI treatment, and of those, 60–80% will complete treatment.
(6)	Treatment of drug-susceptible LTBI reduces the rate of incident active TB due to reactivation; a full course of rifampicin-based TPT is 43–89% effective in preventing incident active TB [29]. Incomplete TPT provides partial protection. Treatment for LTBI caused by drug-resistant *Mycobacterium tuberculosis* provides no protection.
(7)	The benefits of TPT accrue regardless of the outcome of the asylum application and the duration of stay.
(8)	Health benefits considered for this study are individual-level benefits and do not include indirect benefits arising due to onward transmission. The incremental value of these indirect benefits is explored at secondary analysis.

### Model structure and transition probabilities

**Fig. 2:**
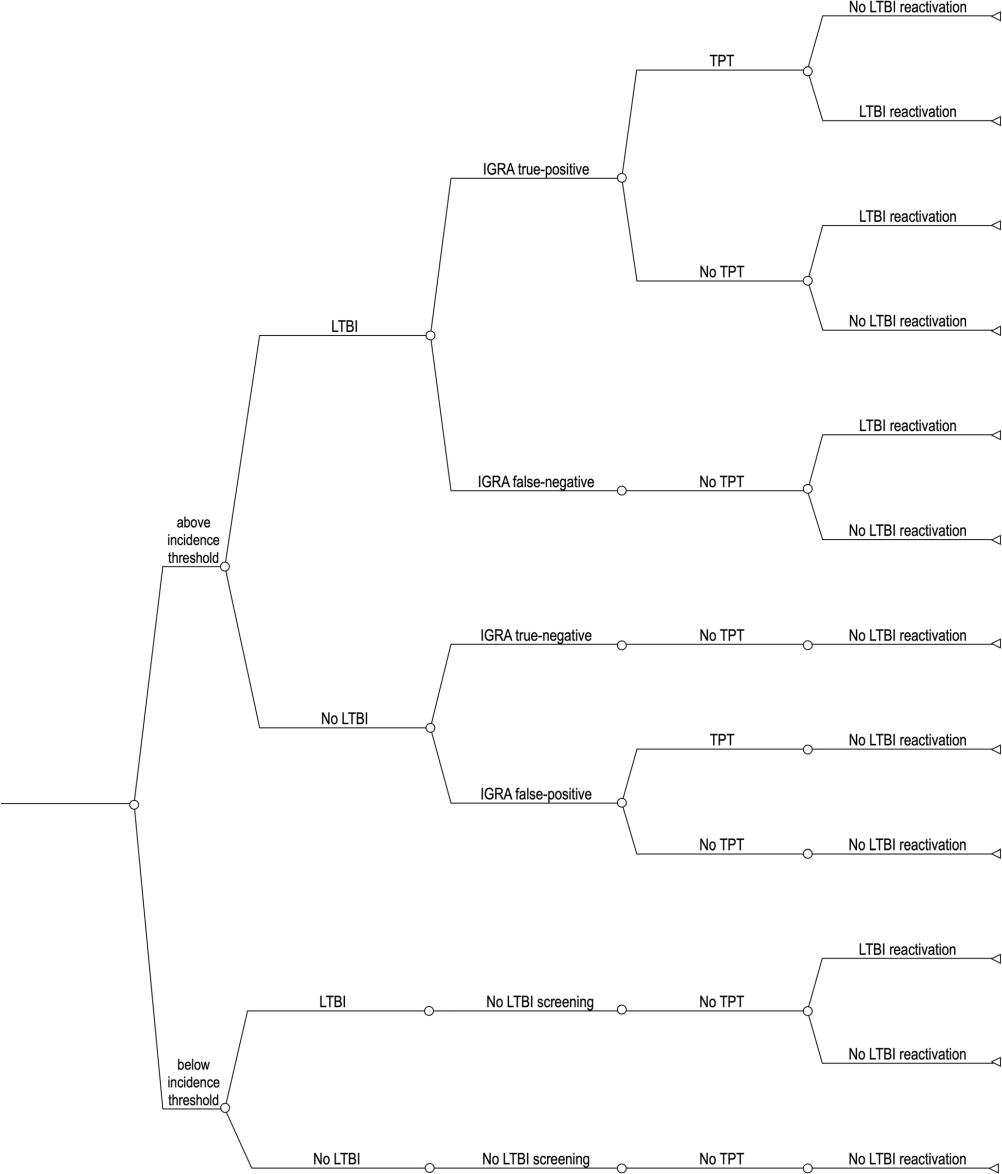
Structure of the decision-tree model. For simplicity, the decision tree shown does not include incomplete tuberculosis preventive treatment (TPT) and TPT for rifampicin-resistant latent TB infection (LTBI)

A schematic of the decision-tree model is shown in Fig. 2. For a given threshold of country-of-origin TB incidence, the model divides the sample population into sub-populations above the TB incidence threshold, eligible for LTBI screening, and below (not eligible). The model then uses transition probabilities for LTBI, positive and negative IGRA test results, TPT initiation and completion, and LTBI reactivation to estimate the number of individuals who develop incident TB post-immigration. We sampled multiple random sets of transition probabilities from ranges derived from the published literature, focusing primarily on studies conducted in Germany and other countries of the European Union.

**Fig. 3:**
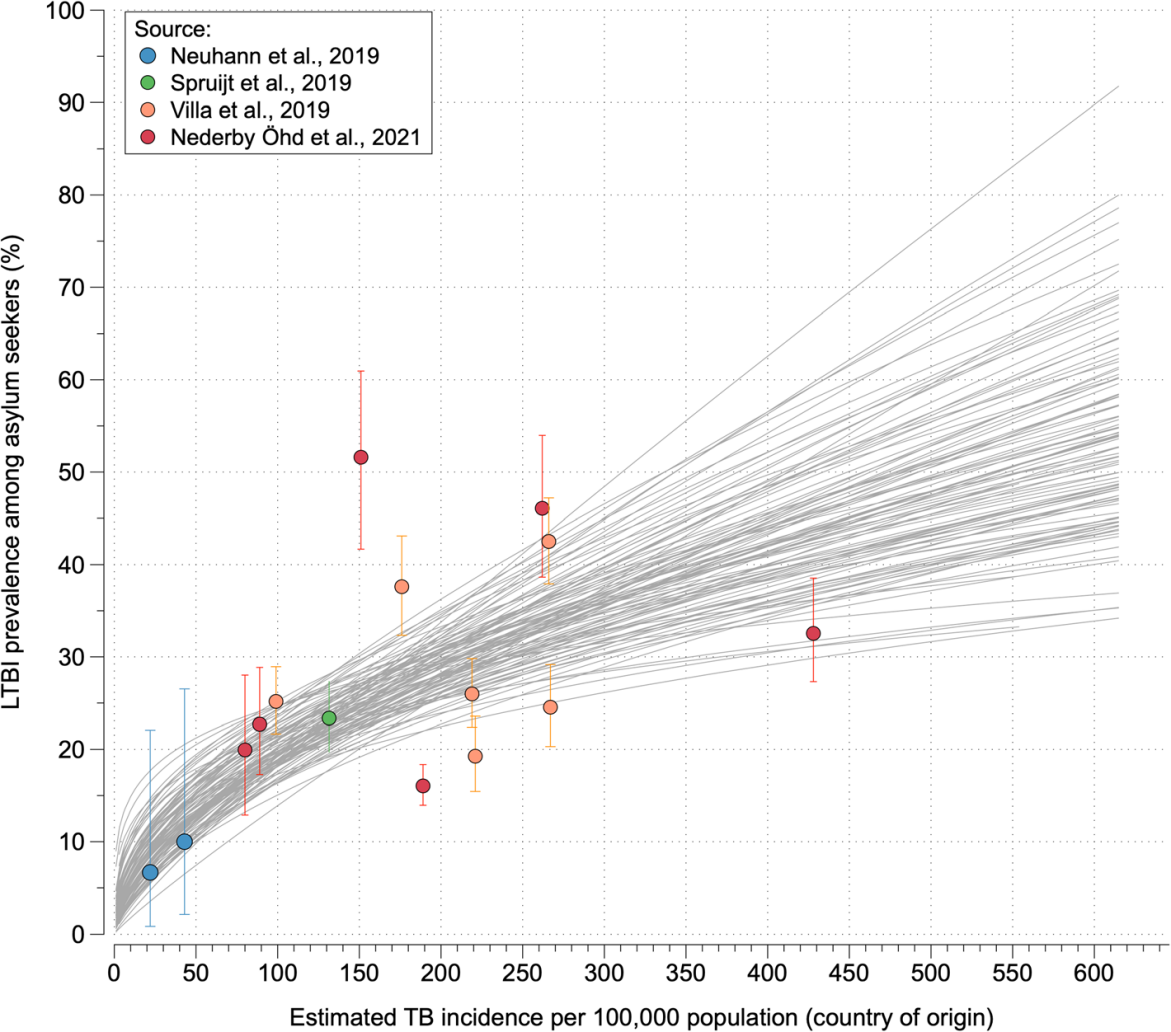
Modeled projections of asylum seekers’ LTBI prevalence as a function of estimated TB incidence in their countries of origin. Colored data points show IGRA-based estimates of LTBI prevalence among asylum seekers obtained from studies in four European countries (Germany, Italy, Sweden, and The Netherlands) with error bars denoting 95% confidence intervals. Grey lines show fitted projections of LTBI prevalence by country-of-origin TB incidence for 100 randomly selected model iterations

Data about the prevalence of LTBI among asylum seekers arriving in Germany and other European countries are limited to date. We considered findings from an earlier literature review published in 2010 [30] and, in addition, reviewed the published literature using PubMed for IGRA-based studies reporting estimates of prevalent LTBI stratified by country of origin among asylum seekers upon-entry to destination countries in Europe. We identified four studies, from Germany, Italy, Sweden, and the Netherlands [23–26]. To obtain estimates of the variation in LTBI prevalence by country-of-origin TB incidence, we fitted a log-log transformed linear regression model to the observed data. For each model iteration in the present study, a coefficient was sampled from the regression model. Country-specific estimates of LTBI prevalence for the sampled asylum seeker population were then derived from the regression model. Figure 3 shows an overview of study data and 100 random model trajectories obtained from fitting the regression model to the estimates of LTBI prevalence obtained from the literature.

IGRA results among asylum seekers with and without LTBI depend on test accuracy. We obtained estimates for IGRA sensitivity and specificity from two meta-analyses [31, 32]. We assumed that asylum seekers with a positive IGRA result and in whom no clinical or radiological evidence for TB disease is found are offered TPT. Given the known challenges of successfully providing healthcare services to immigrant populations [33, 34], we assumed conservative TPT initiation (60–80%) and completion (60–80% of those on treatment [33]) probabilities. We allowed the efficacy of rifampicin-based TPT in reducing LTBI reactivation to vary between 43 and 89%, a range that is based on a recent network meta-analysis [29]. We assumed that incomplete TPT would result in reduced effectiveness. We also accounted for the possibility of drug-resistant *M. tb* infection among asylum seekers with probabilities similar to those estimated for individuals with active TB in the countries of origin. No preventive effect of TPT was assumed for individuals infected with rifampicin-resistant *M. tb*. Table 1 shows parameters and sampling distributions used for the model.

**Table 1:** Model parameters

DESCRIPTION	MEAN VALUE	UNCERTAINTY INTERVAL	SOURCE	PROBABILITY DISTRIBUTION
*Epidemiological & treatment parameters*				
Prevalence of LTBI at entry	Varying^*^	–	[14, 23–25]	–
Sensitivity of IGRA	0.800	0.750–0.840	[31]	beta
Specificity of IGRA	0.980	0.870–0.990	[32]	beta
Probability of TPT initiation	0.700	0.600–0.800	[24, 26, 33]	uniform
Probability of TPT completion	0.700	0.600–0.800	[33]	uniform
Probability of reactivation of untreated LTBI	0.053	0.025–0.080	[11, 27, 28]	uniform
Effectiveness of complete TPT	0.660	0.430–0.890	[29]	uniform
Effectiveness of incomplete TPT	0.250	0.150–0.350	Assumption	uniform
Effectiveness of TPT for rifampicin-resistant LTBI	0	–	Assumption	–
Probability of TPT drug-toxicity events not requiring hospitalization	0.020	0.010–0.030	[35, 36]	beta
Probability of TPT drug-toxicity events requiring hospitalization	0.0003	0.0001–0.0006	[36]	beta
*Cost parameters*				
Cost for performing IGRA incl. Laboratory fees (€)	47.03	37.62–56.43	[37]	uniform
Cost for counselling an IGRA-positive individual for TPT (€)	27.34	21.87–32.80	[37]	uniform
Cost for physician consultation during TPT (fee for 2 quarters of a year; €)	34.72	27.78–41.67	[37]	uniform
Cost for laboratory tests prior to and during TPT (€)	16.18	12.95–19.42	[37]	uniform
Cost for 4-months of rifampicin-based TPT (€)	330.39	322.91–337.87	[37, 38]	uniform
Factor for discounting cost for incomplete TPT	0.375	0.250–0.500	[35]	uniform
In-patient management of drug adverse events of TPT (€)	2553.10	1702.07 – 3404.13	[39]	uniform
Average costs for managing TB disease (€)	8947.65	7158.12 - 10,737.17	[37]	uniform
*Quality-of-life weights*				
Active TB	0.67		[40–43]	–
LTBI	1.00		N/A	–
TPT-related drug toxicity not requiring hospitalization	0.75		[44–46]	–
TPT-related drug toxicity requiring hospitalization	0.50		[44, 47]	–
Other parameters				
Average age at immigration	24		[17]	–
Life expectancy at immigration (years)	59		Estimated from [48]	–
Average time to LTBI reactivation (years)	7.0	6.0–8.0	Estimated from [11]	uniform
TB case-fatality ratio	0.015	0.010–0.020	Estimated from program data	uniform

* Modelled using results from observational studies (see main text)

#### Cost-effectiveness analysis

We estimated total costs under the base-case scenario (no LTBI screening, no TPT) and each incidence threshold scenario in 2020 Euro (€), adopting a German healthcare-system perspective. Cost estimates reflect resources for administering and processing IGRA-based tests, counselling and provision of TPT, and management of TPT-related adverse events in the German public healthcare system (Table 1). We did not account for costs incurred for excluding TB disease among IGRA-positive individuals as we assumed that mandatory clinical and CXR examination remained in place for all asylum seekers aged 15 years and above as per routine policy. Cost estimates were derived from a recent comprehensive costing study that estimated the cost of non-multidrug-resistant TB disease and contact investigation in Germany [37], and other studies (Table 1). All cost estimates were adjusted for inflation using average annual German gross domestic product deflator rates [49]. Estimated total costs for LTBI screening and TPT were offset by discounted future savings of TB diagnostic and treatment costs for two-third of asylum seekers assumed to be still in the country when developing incident TB. This proportion is based on the rate of asylum seekers in 2017–2019 who were granted asylum/permitted to stay in Germany [50].

We estimated the health impact of LTBI screening and TPT by calculating quality-adjusted life years (QALYs) that would be gained through the intervention [51]. QALYs estimated in this study reflect gains in life years and health-related quality of life due to the prevention of TB, and losses in quality of life experienced due to TPT-related drug toxicity events (Table 1). Future costs and health benefits were discounted at an annual rate of 3.0%. To account for the uncertainty around parameter estimates, we ran a total of 1000 Monte Carlo simulations [52] in which multiple sets of parameters were sampled from the pre-specified parameter distributions. We followed the Consolidated Health Economic Evaluation Reporting Standards [53] to report the results of our study. Best estimates of costs and health impact were calculated as the mean, and 95% uncertainty intervals calculated as the 2.5th and 97.5th percentile of resultant simulations.

We estimated incremental cost-effectiveness ratios (ICERs) denoting the extra costs incurred per additional QALY gained for a particular incidence threshold, with respect to the next higher incidence threshold. ICERs are thus equivalent to the incremental cost per QALY gained for a specific stratum of country-of-origin TB incidence (e.g. 150–200 per 100,000) compared to no LTBI screening at all. Germany does not specify a single threshold for the cost per QALY gained to be considered cost-effective [54]. We estimated the probability of LTBI and TPT to be cost-effective for a range of willingness-to-pay (WTP) thresholds including ~€81,300 ($91,447), equivalent to twice the 2020 gross domestic product (GDP) per capita for Germany [49], and international benchmarks including ~€34,000 (£30,000) currently recommended by the National Institute for Health and Care Excellence (NICE) in the United Kingdom, and ~ €87,600 ($100,000), a value that is frequently utilized for health-economic analysis in the United States. Probabilities of cost-effectiveness at a given WTP threshold were calculated as the proportion of model iterations with an ICER estimate not exceeding this threshold [55].

### Sensitivity and scenario analyses

We conducted one-way sensitivity analyses to assess how sensitive our results were to the specified input parameter ranges. Sensitivity analysis refers to the incremental cost-effectiveness of introducing LTBI screening and TPT at an incidence threshold of ≥150 per 100,000 country-of-origin TB incidence.

While the primary analysis does not take transmission into account, we conducted secondary analysis for which we considered additional health benefits to accrue from the prevention of onward transmission from asylum seekers with LTBI reactivation. We assumed a simple scenario in which secondary TB would arise in contacts of equal age compared with the index case (average age at LTBI reactivation: 31 years), an average serial interval of 8–10 years, and similar reductions in quality of life due to TB, and case fatality, compared with those estimated for the index case (see Table 1). We considered variable average numbers of secondary cases ranging from 0.1 to 5.0 per index case.

## Results

We estimate that among 15- to 34-year-old asylum seekers arriving in Germany in 2022, 17.5% (95% uncertainty interval: 14.2–21.6%) will be latently infected with *M. tb*, equivalent to 6597 (4874 - 8832) individuals. Of these, 346 (159–592) will develop incident TB due to the reactivation of LTBI post-immigration.

**Fig. 4:**
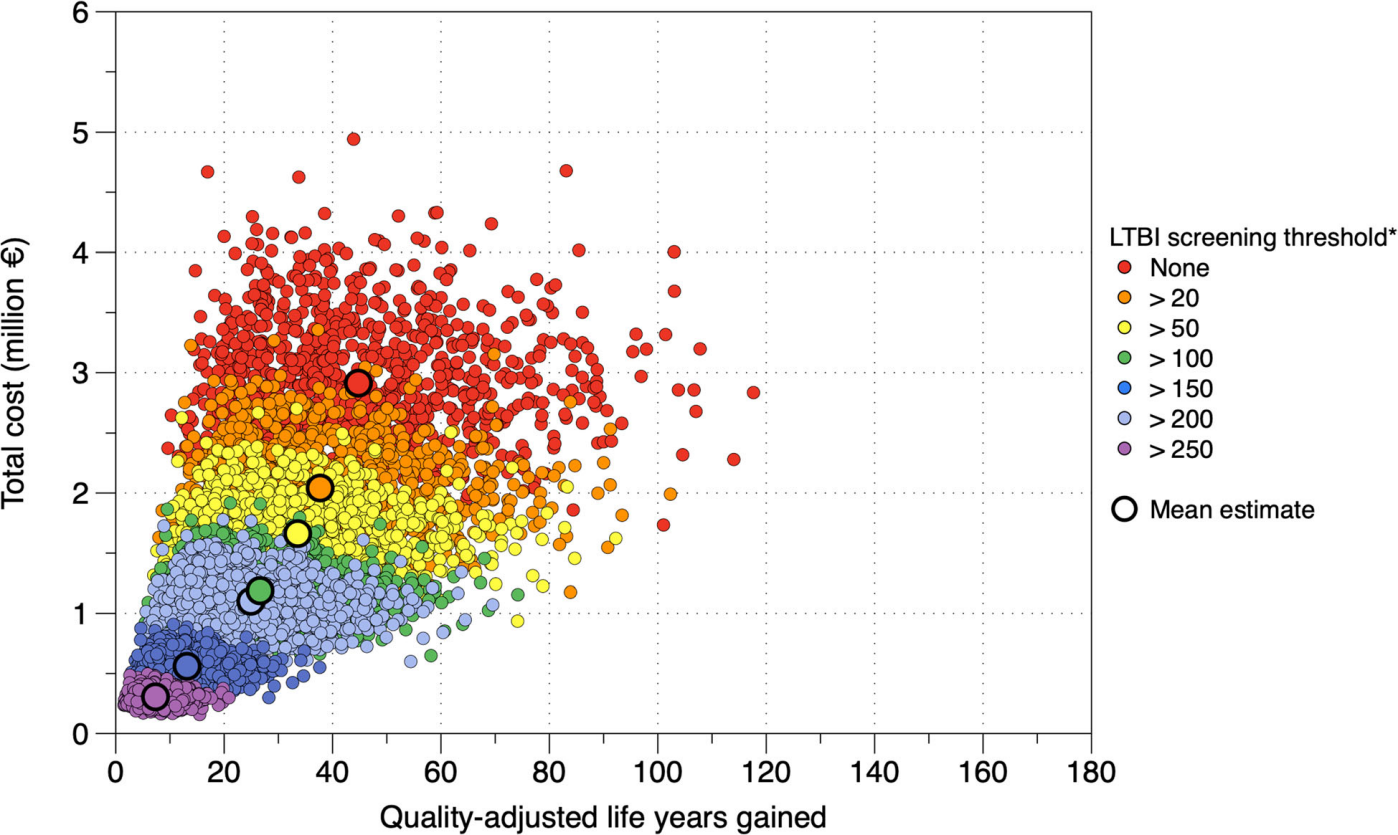
Total cost and quality-adjusted life years gained through screening and treatment for LTBI among 15- to 34-year-old asylum seekers arriving in Germany in 2022. Small circles show single model iterations; large circles denote mean estimates. *The incidence threshold denotes the country-of-origin TB incidence level above which asylum seekers would become eligible for LTBI screening and TPT

An overview of estimated health-system costs, TB cases prevented, and QALYs gained under different incidence thresholds at which asylum seekers would become eligible for LTBI screening and TPT is shown in Table 2 and Fig. 4. We estimate that introducing LTBI screening and TPT at a threshold of 250 TB cases per 100,000 population (country-of-origin TB incidence) would cost €0.31 (€0.20 - €0.42) million, prevent 16 (7–32) TB cases, and 7.3 (2.7–14.8) QALYs would be gained at a cost of €51,000 (€18,000 - €114,100) per QALY. Lowering the threshold to 200 would cost an incremental €53,300 (€19,100 - €122,500) per additional QALY gained relative to the ≥250 threshold scenario - the incremental cost per additional QALY gained would be €55,900 (€20,200 - €128,200) and €62,000 (€23,200 - €142,000) for the ≥150 and ≥ 100 thresholds, respectively, using the next higher threshold level as a reference (Table 2). We estimated that the additional cost per additional QALY gained will be considerably higher at incidence thresholds lower than 100 per 100,000 population (Table 2).

**Table 2:** Estimated costs, TB cases prevented and cost-effectiveness for modeled scenarios of screening and treatment for latent tuberculosis infection among 15- to 34-year-old asylum seekers in Germany, 2022

LTBI screening threshold	Total costs* (million €)	TB cases prevented	QALYs gained	Incr. costs* (million €)	Incr. TB cases prevented	Incr. QALYs gained	ICER (Thsd. € per TB case prevented)	ICER (Thsd. € per QALY gained
≥ 250	0.31 (0.20–0.42)	16 (7–32)	7.3 (2.7–14.8)	0.31 (0.20–0.42)	16 (7–32)	7.3 (2.7–14.8)	22.3 (8.2–50.0)	51.0 (18.0–114.1)
≥ 200	0.56 (0.38–0.77)	29 (12–56)	13.2 (4.9–26.3)	0.25 (0.17–0.35)	13 (5–25)	5.8 (2.1–11.6)	23.3 (8.6–52.2)	53.3 (19.1–122.5)
≥ 150	1.10 (0.74–1.52)	56 (23–105)	24.9 (9.3–49.9)	0.54 (0.37–0.74)	26 (11–50)	11.8 (4.4–23.7)	24.5 (9.2–53.7)	55.9 (20.2–128.2)
≥ 100	1.19 (0.80–1.63)	60 (24–113)	26.6 (9.9–53.5)	0.09 (0.06–0.12)	4 (2–7)	1.7 (0.6–3.3)	27.1 (10.5–59.8)	62.0 (23.2–142.0)
≥ 50	1.66 (1.13–2.27)	75 (31–142)	33.6 (12.5–67.1)	0.47 (0.33–0.66)	16 (6–30)	6.9 (2.6–13.8)	36.0 (14.7–78.4)	82.4 (31.6–184.7)
≥ 20	2.04 (1.39–2.78)	84 (34–157)	37.7 (14.0–74.7)	0.38 (0.26–0.54)	9 (4–18)	4.1 (1.5–8.7)	48.7 (19.8–106.7)	111.8 (42.7–251.9)
None^†^	2.91 (2.02–4.02)	100 (41–187)	44.8 (16.8–88.3)	0.87 (0.60–1.25)	16 (5–35)	7.1 (2.2–16.3)	68.0 (26.1–158.7)	156.3 (54.4–373.3)

Intervals in brackets denote 95% uncertainty intervals. Latent tuberculosis infection (LTBI) screening thresholds shown denote levels of country-of-origin tuberculosis incidence above which asylum seekers would be eligible for LTBI screening and tuberculosis preventive treatment. Screening threshold alternatives are presented in the order of increasing cost, starting with the least costly screening scenario (≥250 incidence threshold). Increments for tuberculosis cases prevented, quality-adjusted life years (QALYs) gained and incremental cost-effectiveness ratios (ICER) were calculated with respect to the previous less costly alternative (i.e. ≥250 threshold compared to no screening, each of the other thresholds compared to the next higher threshold, “none” compared to the ≥20 threshold). *Costs incurred for LTBI screening and TPT are offset for discounted future savings of costs for the management of TB among those in whom TB was prevented - see main text. † No threshold was used; all individuals were eligible for screening regardless of country-of-origin TB incidence

The highest incremental health benefit was estimated for the ≥150 incidence threshold. Here, 11.8 (4.4–23.7) additional QALYs would be gained at marginally higher incremental cost per QALY, relative to the ≥200 incidence threshold. The same threshold would allow to detect an estimated 44% (35–52%) of all LTBIs and prevent 16% (10–23%) of incident TB cases expected among all 15- to 34-year-old asylum seekers in the absence of LTBI screening/TPT.

**Fig. 5:**
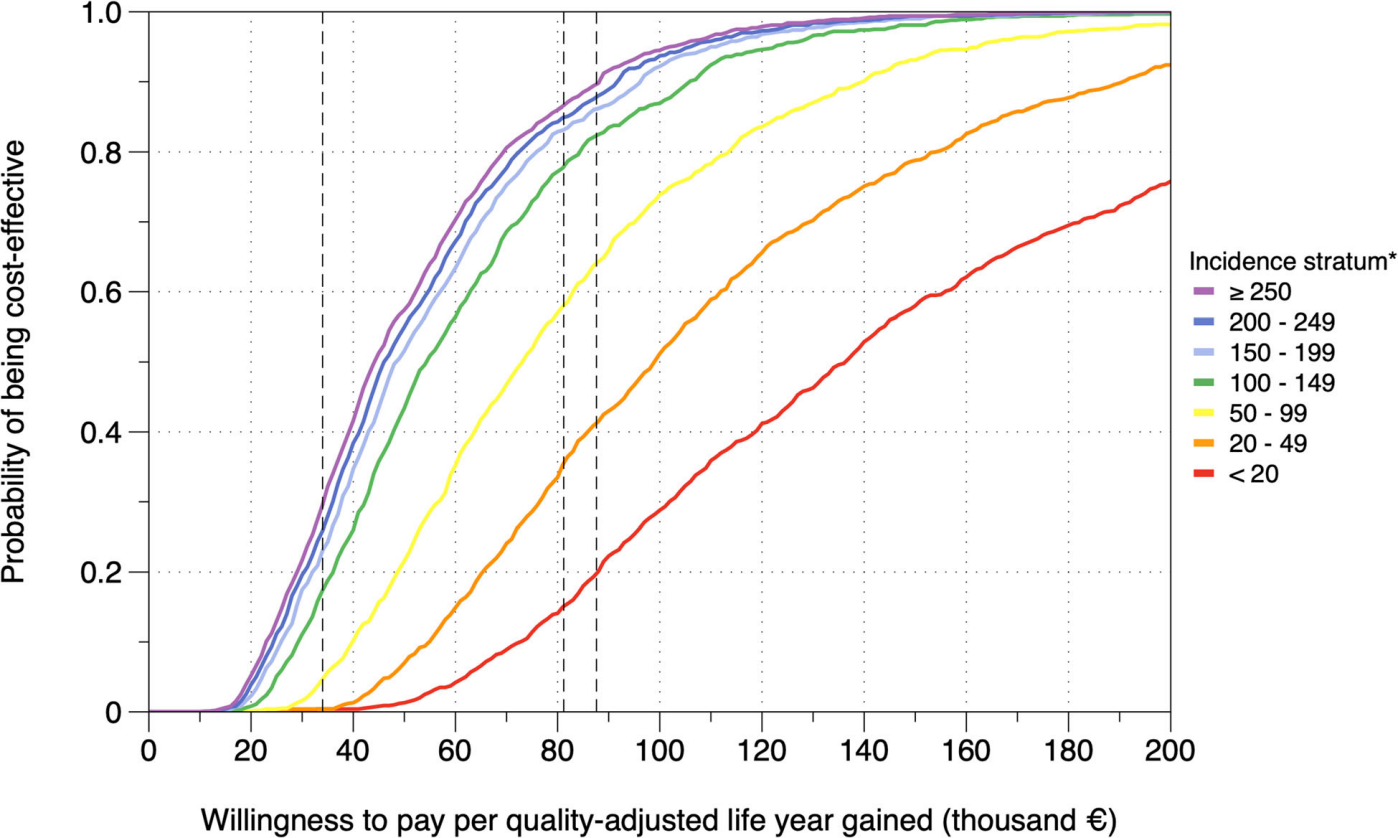
Probabilities of LTBI screening and preventive treatment being cost-effective at different thresholds of willingness to pay per quality-adjusted life year gained. The dashed lines denote the following benchmark thresholds for cost-effectiveness (from left to right): £30,000 (~€34,000) – a threshold recommended by the National Institute for Health and Care Excellence (NICE) in the United Kingdom; €81,300 ($91,447), equivalent to twice the 2020 gross domestic product (GDP) per capita for Germany; ~€87,600 ($100,000) – a threshold that is frequently utilized for health-economic analysis in the United States. *Incidence strata denote strata of asylum seekers by estimated TB incidence in their countries of origin

If healthcare services were willing to pay an extra €81,100 per additional QALY gained (~twice the 2020 GDP per capita for Germany), an incidence threshold ≥250 would have a probability of 87% of being cost-effective, relative to no LTBI screening / no TPT (Fig. 5). For the same willingness to pay, lowering the incidence threshold to ≥200, ≥150, or ≥ 100, would have a probability of 85%, 83%, or 78%, respectively, of being cost-effective, with respect to the next higher incidence threshold. Probabilities of cost-effectiveness were considerably lower for asylum seekers below 100 TB incidence (Fig. 5).

**Fig. 6:**
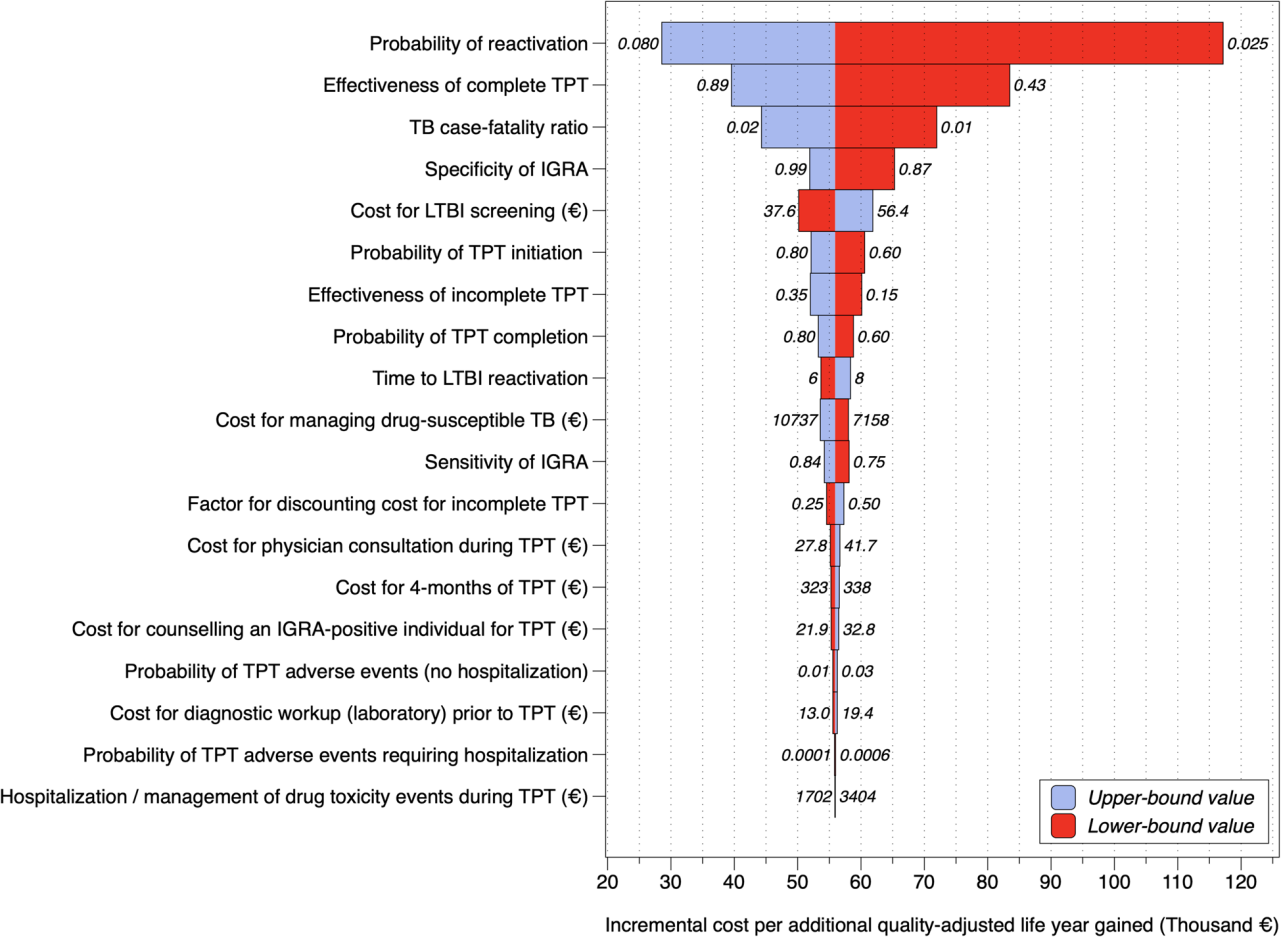
Sensitivity analysis of incremental cost-effectiveness ratio for the ≥150 incidence threshold. The bars show the distance between the low or high estimate and the best estimate obtained from probabilistic analysis (€ 55,911; see Table 2); parameters are sorted from high to low absolute difference to the probabilistic estimate; labels outside of bars denote the upper or lower-bound parameter value investigated

The incremental cost per additional TB case prevented at the ≥150 incidence threshold was most sensitive to the probability of LTBI reactivation, the effectiveness of TPT, the TB case-fatality ratio, the specificity of IGRA, and the cost for LTBI screening (Fig. 6).

**Fig. 7:**
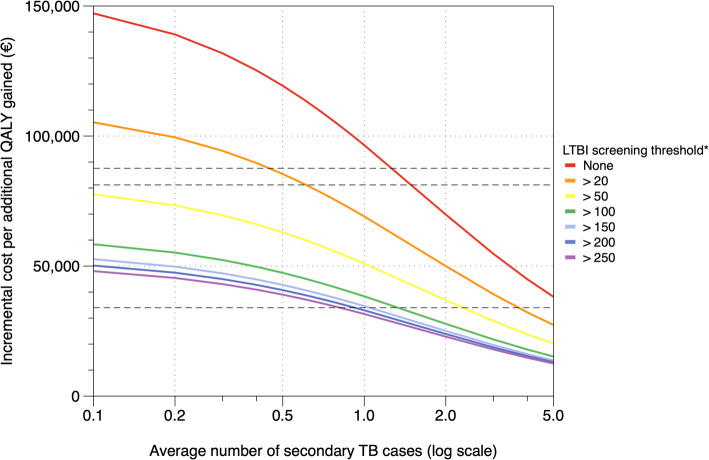
Secondary analysis: incremental cost-effectiveness ratios by average numbers of secondary TB cases. The dashed lines denote the following benchmark thresholds for cost-effectiveness (from top to bottom): ~€87,600 ($100,000) – a threshold frequently utilized for health-economic analysis in the United States; ~€81,300 ($91,447), equivalent to twice the 2020 gross domestic product (GDP) per capita for Germany; ~€34,000 (£30,000) – a threshold recommended by the National Institute for Health and Care Excellence (NICE) in the United Kingdom; *The incidence threshold denotes the country-of-origin TB incidence level above which asylum seekers would become eligible for LTBI screening and TPT

Secondary analysis showed the extent to which incremental cost-effectiveness ratios decreased with the average number of secondary TB cases arising from an asylum seeker with TB due to LTBI reactivation (Fig. 7). We estimate that with one secondary TB case for every five LTBI reactivations (average number: 0.2), incidence thresholds above 100 (or higher) resulted in less than €55,200 incremental cost per additional QALY gained, relative to the next higher incidence threshold.

## Discussion

In this study, we estimated health benefits and costs of introducing LTBI screening and TPT among 15- to 34-year-old asylum seekers upon entry to Germany. We explored the cost-effectiveness of this intervention at different eligibility thresholds of country-of-origin TB incidence.

Our analysis suggests high costs per QALY gained among individuals from countries with a lower incidence threshold (i.e. lower than 100 per 100,000) suggesting that LTBI screening and TPT are unlikely to be cost-effective unless targeted to asylum seekers from countries with higher TB incidence, consistent with findings from a similar analysis in the United Kingdom [14]. The higher incremental cost per additional QALY gained among individuals below the 100-incidence threshold are due to the relatively low prevalence of LTBI expected in this group. Introducing LTBI screening and TPT at higher incidence thresholds could yield individual health benefits at reasonable costs per QALY gained. We estimate that limiting LTBI screening to individuals from countries with a TB incidence ≥250 per 100,000 population would cost €22,300 per TB case prevented, consistent with an earlier estimate from the United Kingdom (£20,819 ~ €23,600) [14], and €51,000 per QALY gained. Lowering the threshold to ≥150 TB incidence would more than triple the health impact (QALYs gained) compared to the ≥250 incidence threshold, with relatively low incremental costs incurred per QALY (Table 2).

Our estimates of health benefits and costs represent conservative estimates that do not take into account additional health benefits and savings due to reductions in onward transmission of *M. tb*, for example to child and adult household contacts. Secondary analyses in which we recalculated incremental cost-effectiveness ratios for varying average numbers of secondary TB cases showed that these indirect benefits could be relevant for determining cost-effectiveness of LTBI screening and TPT among asylum seekers. In an earlier study conducted in Berlin [56], we found a higher rate of incident TB among first and second generation immigrants than in the native resident population, consistent with transmission occurring within migrant populations.

We assumed that LTBI screening and TPT would be offered in addition to clinical and CXR-based examinations that are currently mandatory for all asylum seekers aged 15 years and older upon-entry to Germany. Whether the current status quo of screening for TB disease is cost-effective for TB, and whether LTBI screening and TPT should therefore complement or replace the disease-based screening approach is currently not known. A recent modelling study of CXR-based screening in one of the German federal states [38] suggested a country-of-origin TB incidence of 50 per 100,000 population as a reasonable threshold based on substantially higher costs for TB cases found and prevented among individuals from countries with a lower TB incidence. Assuming screening was limited to individuals above the 50-incidence threshold, the estimated costs per TB cases detected through screening and per secondary TB case prevented were €21,704 and €84,003, respectively [38]. In our study, we estimated an average cost of €26,255 per TB case prevented above the 50-incidence threshold, notably without taking onward transmission into account.

Low rates of initiating and completing preventive treatment are important concerns ahead of introducing LTBI screening among asylum seekers [33] as not every individual with confirmed LTBI will be eligible, willing to start and able to complete TPT. To account for these challenges, we specified conservative estimates of TPT initiation (60–80% of those IGRA-positive) and treatment completion (60–80% of those initiating TPT). Sensitivity analysis showed that variation within these ranges had moderate impact on estimated incremental costs per additional QALY gained. Nevertheless, the effects and cost-effectiveness of LTBI screening will be conditional on reasonable uptake of and retention in care post-immigration. Anticipated challenges in delivering healthcare interventions to asylum seekers may also impact the success of the current CXR-based screening strategy. For example, a large study of screening in four reception centres in Germany showed that one-third of asylum seekers with CXR results suggestive of active TB were lost to follow-up, considerably reducing the beneficial effect of screening [57].

Our study constitutes a first step towards a better understanding of the benefits and costs of LTBI screening and TPT among asylum seekers in Germany, a country that traditionally relies on CXR-based screening for TB disease. We note the following limitations.

Our model is based on numbers of asylum seekers registered in Germany in recent years, their distribution by country-of-origin TB incidence, and recent TB-related cost estimates specific for Germany. There is currently a lack of country-specific data about the prevalence of LTBI, the rate of LTBI reactivation, losses in quality of life due to TB and case fatality among asylum seekers. Given the sparsity of studies and data in the German context, we relied, where necessary, on estimates from studies conducted elsewhere in Europe. There is considerable uncertainty around parameter estimates. We accounted for this uncertainty by specifying wide uncertainty ranges at probabilistic analysis. We note that our model could be easily updated to consider estimates from future research necessary to improve the validity and precision of the parameter estimates.

Total numbers of asylum seekers considered for this study reflect declining trends observed in the years prior to the COVID-19 pandemic. Lockdowns, travel restrictions and suspensions of asylum-related activities during the COVID-19 pandemic have resulted in substantial additional decreases in asylum applications in Germany and other European countries [58] with considerable uncertainty for projections in the forthcoming years. Our analysis refers to a post-COVID phase and assumes that COVID-related reductions are temporary, i.e. that immigration in the year 2022 will return to trends observed prior to the COVID-19 pandemic. While absolute costs and QALYs gained estimated in this study should be interpreted with caution as they depend on total numbers of asylum seekers, we don’t think that relative estimates of costs per health impact will be affected.

We modelled variation in LTBI prevalence by country of origin, taking several European studies into account. We note considerable heterogeneity in LTBI prevalence estimates within and between these studies, suggesting that factors other than country of origin have contributed to the observed prevalence of LTBI among asylum seekers. WHO estimates of country-level TB incidence may not accurately reflect the risk of LTBI among asylum seekers; infection may also happen after leaving the country of origin [59]. While uncertainty in the fitted model refers to the (average) trajectory of LTBI prevalence by country-of-origin TB incidence, it does not necessarily capture the additional heterogeneity observed between studies.

Finally, we focused our analysis on asylum seekers aged 15 to 34 years. We did not include children and young adolescents aged < 15 years since their evaluation for *M. tb* infection via IGRA or TST is already recommended as part of their screening for TB disease in Germany [60]. Also, there is particular uncertainty around the proportion of children who arrive with LTBI and their rate of LTBI reactivation and associated health impact. However, results from a study among unaccompanied minor refugees in Germany [61] showed that IGRA-based screening and preventive treatment were feasible and well tolerated, suggesting that policies of LTBI screening and TPT among asylum seekers may include children.

## Conclusions

Our findings suggest that introducing LTBI screening and TPT among 15- to 34-year-old asylum seekers upon entry to Germany could produce health benefits and reasonable costs if targeted to individuals from countries with a high TB incidence. An incidence threshold of 150 per 100,000 population would allow for the detection of a reasonable fraction of LTBI and produce considerable health benefits at low incremental costs compared to higher thresholds. Additional empirical research is needed to improve our estimates and provide further guidance for policy making. This includes studies of LTBI prevalence among asylum seekers upon entry, stratified by country-of-origin TB incidence, and the rate of TB in the years post-entry. Our results support the conduct of pragmatic trials to assess the feasibility, effectiveness and cost-effectiveness of LTBI screening and TPT among asylum seekers. Alternative short-term preventive treatment regimens, such as rifapentine/isoniazid daily for 1 month [62] or weekly for 3 months [63], could be considered once rifapentine is approved for use in the European Union. Risk categories other than country of origin could be considered for screening policies to identify those asylum seekers at highest risk of LTBI and TB disease [64] who would benefit the most from TPT. Operational research is also needed to systematically evaluate the coverage, effectiveness and costs of the current CXR-based screening policy, including the health outcomes of asylum seekers who test positive at screening.

If well implemented, LTBI screening and TPT among asylum seekers could complement current efforts to address and reduce the risk of TB among immigrants [56] and other high-risk groups in Germany, thus ensuring continued progress towards TB elimination in the forthcoming years.

## Data Availability

The dataset with asylum statistics used for sampling asylum seeker populations for Germany is publicly available from *Eurostat*, the statistical office of the European Union (https://ec.europa.eu/eurostat/). Other data/estimates used for the decision-analytic model were obtained from the published literature.
